# Endoscopic ultrasound gallbladder drainage (EUS-GBD) with LAMS: While we know how to drain we are still questioning who to drain

**DOI:** 10.1055/a-2487-7723

**Published:** 2025-01-13

**Authors:** Andrea Lisotti

**Affiliations:** 1 9296 Gastroenterology Unit, Hospital of Imola, University of Bologna, Imola, Italy

**Keywords:** Endoscopic ultrasonography, Biliary tract, Intervention EUS



Acute cholecystitis (AC) is a complication frequently reported by patients suffering from symptomatic biliary stone disease. International guidelines agree regarding the indication for laparoscopic cholecystectomy in AC patients fit for surgery. On the other hand, in the last decade, an increasing amount of knowledge has supported the recommendation for gallbladder drainage in case of severe AC or high-risk surgical patients 1 .

Among different available strategies, endoscopic ultrasound-guided gallbladder drainage (EUS-GBD) presents better technical and clinical outcomes compared with endoscopic transpapillary gallbladder drainage (ET-GBD) 2 . Moreover, the DRAC-1 randomized controlled trial demonstrated that despite similar success rates and safety profile, EUS-GBD is superior compared with percutaneous transhepatic gallbladder drainage (PT-GBD) in terms of long-term outcomes because a dramatic reduction in adverse events (AEs) and recurrent AC has been observed 3 .

In the present issue of Endoscopy International Open, Yakira et al. published the results of a multicenter study involving 18 referral centers in the United States. The authors retrieved data from 110 high-risk surgical patients admitted for AC who underwent EUS-GBD with lumen-apposing metal stent (LAMS) and who had completed at least 1-month follow-up after the procedure 4 . The present study confirms the optimal outcomes observed in a similar population prospectively enrolled in an US multicenter trial by Irani et al. 5 .

In detail, Yakira et al. reported a 99% technical success rate coupled with a 97% clinical success rate; this enthusiastic approach allowed a sustained clinical success in up to 90% of these patients with EUS-GBD. As observed in any rescue strategy for complex clinical scenario, EUS-GBD also is burdened by suboptimal outcomes and long-term clinical failures. Patients who underwent EUS-GBD with LAMS present a low incidence of recurrent AC (range 2%-4%); however, a not-negligible amount of stent-related AEs were observed over time (i.e., stent occlusion, food impaction, stent migration, buried LAMS syndrome). Several strategies have been proposed to reduce the incidence of long-term stent dysfunction. Among these strategies, several authors proposed to plan LAMS removal after 4 to 8 weeks, considering substitution with double pigtail plastic stents in case of a large amount of residual stones in the gallbladder.

The present study of Yakira et al. tried to answer to the unsolved issue of the usefulness of LAMS removal. The authors failed to find any improvement in long-term outcomes, both recurrent AC and AEs, in case of LAMS removal 4 . Another large Spanish study recently demonstrated that the strategy of keeping the LAMS in situ over time could be considered a safe option in this setting. Moreover, in this study the authors suggested that the transduodenal approach for EUS-GBD with LAMS seems correlated with a lower incidence of long-term AEs. 6 Indeed, European Society of Gastrointestinal Endoscopy guidelines recommend transduodenal over transgastric approach for EUS-GBD to reduce risk of food impaction and buried LAMS; unfortunately, no robust data are available on which to base a strong recommendation because most evidence came from retrospective studies that were not specifically designed to answer this question 7 .

Although the optimal short-term technical and clinical success rates and all the efforts to optimize the long-term outcomes, we still observe a significant mortality among the fragile population of high-risk surgical patients suffering from AC who undergo EUS-GBD. The results of a previous meta-analysis including nine non-comparative studies analyzing 398 patients who underwent EUS-GBD (with several techniques and different devices) reported a shocking pooled mortality rate of 26% 2 .

Although the first step forward has been taken, thanks to introduction of electrocautery-enhanced LAMS delivery systems for EUS-GBD, the main remaining issue is identification of the best candidates for EUS-GBD. Our first experience with EUS-GBD with LAMS suggested that long-term patient mortality seems to be influenced more by comorbidities than procedure outcomes [8,9.] In detail, presence of significant comorbidities and acute kidney injury were independently related to long-term mortality. We identified a Charlson Comorbidity Index (CCI) cut-off value of 6 to predict long-term survival after EUS-GBD, independent from procedure clinical success rate. This result suggested a local improvement in the EUS-GBD policy; in fact, in our center, we prefer PT-GBD as a primary drainage strategy for patients with severe comorbidities and EUS-GBD in case of CCI ≤ 6. Finally, drainage internalization through conversion from PT-GBD to EUS-GBD is considered to allow long-term management of patients after biliary sepsis resolution 8 .

To date, the crucial question has been how to drain, whereas in the next future, we should first ask ourselves who to drain.

In conclusion, we agree with Yakira et al. that EUS-GBD with LAMS should be considered the destination therapy in this difficult setting, based on the demonstrated successful outcomes. We call for technical and technological advancements together with theoretical improvements to raise the bar of this promising strategy.
